# Study on Relationship Between Mechanical Properties and Water Absorption Characteristics of Mortars by Using Digital Image Correlation Method (DICM)

**DOI:** 10.3390/ma18051182

**Published:** 2025-03-06

**Authors:** Muhammad Usman, Hikaru Nakamura, Muhammad Shoaib Karam, Taito Miura, Go Igarashi

**Affiliations:** 1 Department of Civil and Environmental Engineering, Nagoya University, Furo-cho, Chikusa-ku, Nagoya 464-8603, Japan; t.miura@civil.nagoya-u.ac.jp; 2 Department of Civil Engineering, University of Engineering and Technology, Lahore, Narowal Campus, Narowal 15601, Pakistan; shoaib.karam@uet.edu.pk; 3 Department of Environmental Engineering and Architecture, Nagoya University, Furo-cho, Chikusa-ku, Nagoya 464-8603, Japan; go.igarashi.vj@gmail.com

**Keywords:** water absorption, DICM, strain, expansion, concrete materials

## Abstract

The evaluation of water absorption in concrete is regarded as an important indicator for assessing the causes of its deterioration and durability. Traditionally adopted methods for durability assessment in concrete materials, however, lack in providing real-time monitoring of the absorption process and information about the material deformability at surfaces (volumetric changes) caused by swelling in cement hydrates (i.e., C-S-H). In this study, a one-dimensional water absorption test was performed on small-size mortar specimens of three different strengths, and their top (flattened) surface was continuously monitored for volume changes by utilizing surface strain gauges along with the DICM. After the water absorption test, the same specimens were tested to determine mechanical properties such as compressive strength and Young’s modulus. Moreover, the water absorption characteristics, like depth/rate, were evaluated in DICM by tracing changes in waterfront positions with the progression of strains during the water absorption process in mortars. Additionally, the surface strain gauges confirmed the accuracy of strains evaluated by the DICM. The absorption characteristics obtained from observations in the DICM were correlated with the mechanical properties and expansion strain of the test specimens. The results indicated that the durability properties were not only related to the water absorption rate but also to the mechanical properties and volume changes due to saturation.

## 1. Introduction

Concrete is the most widely used material in the construction industry, and its worldwide consumption stands second after water [1]. On the other hand, all concrete structures experience durability-related damages and performance degradations even before reaching their design service life, which are caused by chemical and/or physical factors, such as freeze–thaw cycles, carbonation, sulfate/chloride attack, etc. [2,3,4,5]. Water is usually involved in the entire process of these degradations, which is directly involved in all kinds of physical deteriorations, and as a carrier of aggressive ions, it is indirectly involved in the chemical deteriorations of concrete materials/structures [6]. Therefore, water transport in concrete materials is regarded as a representative descriptor to reflect the durability of concrete structures [6], making it crucial to evaluate water transport characteristics in concrete materials and their impact on several physical factors from the perspective of concrete durability.

Moreover, water transport in concrete materials is strongly influenced by the pore volume fractions (i.e., porosity) and the pores’ interconnectivity [7,8,9,10] and has been extensively evaluated, mainly by considering three modes, including capillary absorption [2,9,11,12,13,14,15], diffusion [16,17] and penetration or permeability [18]. However, among these modes, the capillary absorption (also known as sorptivity) is the most commonly used to investigate water transport capacity in concrete materials. Concrete materials are able to take up water under capillary suction, and this ability has been regarded as an important indicator for assessing the causes of deteriorations in concrete materials and devising strategies to control their rates in concrete structures [15,19,20].

Water absorption tests are most extensively conducted to assess the durability of concrete materials [21] by adopting procedures laid out in ASTM C1585 [22]. In this method, the rate of water absorption (sorptivity) in unsaturated concrete materials is determined by repeatedly performing gravimetric measurements during the water absorption process, and a water content versus t^0.5^ plot is obtained, which describes the absorption process as primary (rapid) and secondary (slow) rates of absorption in cement-based concrete materials. The rapid rate of absorption is controlled by capillary absorption, which is further influenced by pore volume fraction and its distribution and dictates the durability of the concrete materials. Meanwhile, the slow rate is associated with the total porosity in the matrix and fails to provide sufficient information related to durability [15,23]. Repeated mass measurements for eight days, especially for the initial six hours, make this method laborious and time-consuming. Apart from this, it lacks in providing information about the spatial distribution of water in saturated regions of concrete [24]. Also, it does not provide any information about deformations at the specimen surface, as reported in some recent studies [25], which is primarily caused by swelling of the cement hydrates, i.e., C-S-H during the water absorption process, as reported in studies [25,26,27,28,29].

Moreover, the commonly used methods to visualize capillary absorption and quantify the water content profiles in concrete materials include nuclear magnetic resonance (NMR) and electrical technology, as well as X-ray computed tomography (XCT), etc. But all of these methods have their own limitations, like the NMR method, which is highly sensitive to water molecules [28,30], and its results can easily be affected by factors such as hydrogen in the calcium–silicate–hydrate (C-S-H) gel; therefore, obtaining spatial distribution information about water through NMR results can be suspicious. Similarly, electrical technology tools like electrical impedance tomography (EIT) [31], electrical resistance tomography (ERT) [32], and electrical impedance spectroscopy (EIS) [33] are used to visualize or evaluate water absorption in concrete with changes in electrical parameters. However, various factors such as chloride content, metal composition, and temperature can affect the test results. Moreover, neutron imaging offers the visualization of water absorption by detecting neutron beam absorption in concrete specimens, and this technique is particularly suitable for testing hydrogen atoms. However, it cannot measure specific ions like paramagnetic ions [20] which are normally present in concrete or cement-based materials. Additionally, it relies on a high-energy source typically available only in synchrotron radiation beamlines, which limits the widespread adoption of this method.

Based on the above discussion, it can be stated that despite the limitations in the accuracy of measurements for quantifying water content in concrete materials, all the methods traditionally adopted to evaluate water absorption characteristics are either laborious, time-consuming, sensitive to concrete qualities, or demand special arrangements. Moreover, they are unable to provide detailed/precise information on possible volume changes caused by deformations in concrete materials (due to swelling in cement hydrates) and cannot provide visualizations of water progression altogether. On the other hand, the Digital Image Correlation Method (DICM), being a robust, non-destructive strain measurement technique, can evaluate concrete deformations with high accuracy and reliability. It can provide strain visualization in the form of strain contours/maps over the regions of interest [34,35,36,37]. Owing to its efficiency in engineering evaluations, the DICM has emerged as the most popular tool among researchers, especially in the past decade. Its use is continuously expanding, not only in mechanical testing on structural elements and composite materials [35,36,38,39,40,41,42,43,44,45], but also in non-destructive evaluations of concrete, for example, corrosion evaluation [46]; studies of the influence of water-to-cement ratios at interfacial transition zones in cement-based materials [47]; shrinkage in cement-based concrete materials [48]; as an approach complementing the results of numerical simulations [49,50], etc. Recently, one of the studies by Dzaye et al. [51] also applied the DICM on freshly poured mortars and succeeded in quantifying settlement and shrinkage after the validation of DIC results through traditional approaches (LVDTs: Linear Variable Displacement Transducers). More specifically, in their pilot study, Igarashi et al. [52] utilized the DICM to evaluate the deformations in concrete caused by the swelling of C-S-H during water absorption after drying up to 105 °C and succeeded in visualizing the progression of strains at the surface of specimens in the mortar parts of the concrete. However, this study was not supported by any validation of strain calculated in the DICM, for example, by strain gauges, etc., like [51], and the effect of surface contrast changes in saturated regions was neglected, as pointed out in the author’s previous study [53]. On the other hand, ensuring good contrast and producing high-quality speckle patterns for tracing and matching are strongly recommended by previous studies [34,37,44,54,55,56] to yield precise results in the DICM. Moreover, in the past, many researchers, e.g., [17,57,58], have also reported the relationship between mechanical properties and water absorption characteristics determined by traditional methods (described above). But in these studies, the evaluated water absorption characteristics included certain assumptions and lacked proper evidence of phenomena during the water absorption process. Moreover, accurate prediction of the service life of concrete structures is an everlasting challenge among researchers because of the involvement of various factors and complexities in the assessment methods.

Therefore, in this context, the research presented in this paper encompasses the relationship between the water absorption characteristics, deformability during absorption (evaluated by real-time monitoring by the simply set Digital Image Correlation Method (DICM)), and the mechanical properties of mortars with three different strengths. At first, a one-dimensional water absorption test was conducted on small-size rectangular specimens of mortars. Throughout the absorption process, the top (flat) surface of the specimen was monitored for strain development by utilizing surface strain gauges, together with capturing the digital images for the DICM. All the specimens were then tested under compression to determine their mechanical properties (i.e., compressive strength, *f_cm_*’ and Young’s modulus, *E_m_*). Accordingly, the validation of strain measurements in DICM was investigated while accounting for the effects of contrast changes in images. At the end, the water absorption characteristics, like absorption depth/rate and material deformability (strains), were evaluated through observations in the DICM and correlated with the mechanical properties of the same specimens.

## 2. Experimental Outlines

The following sections describe the details of all experimental procedures adopted in this research with some improvements in test setup used in the authors’ previous study [53].

### 2.1. Materials and Mix Proportions

The test specimens of mortars with three different strengths (categorized as low, medium, and high quality) based on varying water-to-cement (W/C) ratios were prepared by using high-early-strength cement for low- and medium-strength mortars and ordinary Portland cement (OPC) for high-strength mortars. Two types of commercially available chemical admixtures were used in preparing the mortar mixes: an air-entraining agent (AE) in low- and medium-strength mortars and the superplasticizer (SSP-104) in high-strength mortars to maintain consistency. The details of each mixed proportion are outlined in Table 1.

### 2.2. Test Specimens

This study involved small-size rectangular specimens with three different strengths with nominal dimensions of length (L) = 150 mm, width (W) = 100 mm, and thickness/height (H) = 50 mm. Six specimens in total (two from each strength case) were cast. Table 2 presents the details of each specimen’s measured dimensions, ages at curing, testing (for water absorption and compression), and the compression test results. Regarding the age of curing, water absorption tests were carried out turn by turn. Therefore, differences in curing and testing ages in low and medium specimens appeared. However, the curing duration was kept relatively longer to minimize the effect of different curing ages, in which all the specimens were cured under water inside a temperature- and humidity-controlled room while maintaining relative humidity at 95 ± 5% and room temperature at 23 ± 2 °C for the whole curing duration.

**Figure 1:**
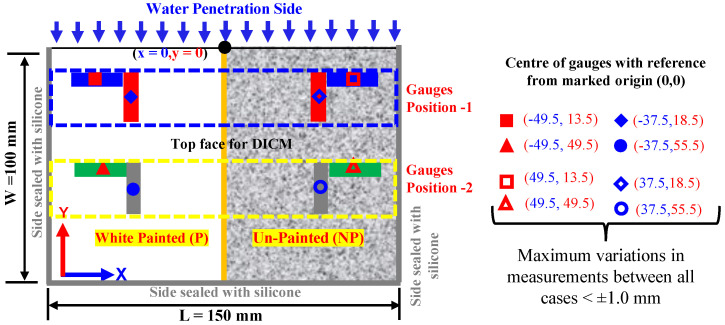
Flat surface (*xy*-plane with nominal dimensions) of the test specimen showing white-painted (P) and unpainted (NP) halves and the positioning of surface strain gauges with respect to water absorption direction.

**Figure 2:**
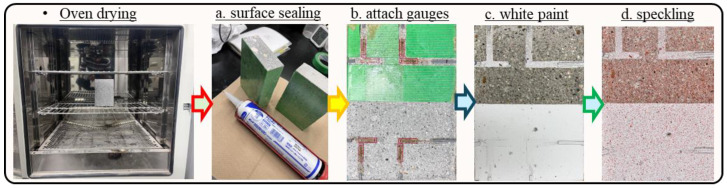
Specimen preparation for DICM with one-dimensional water absorption test.

Irrespective of the differences in mix compositions and the curing conditions/ages, the presence of initial moisture content in the cement-based materials has considerable influence on their water absorption characteristics [2,59], and it can lead to misunderstandings of actual absorption behavior and the absorption rates, etc. [2,8,14,22,23,59,60]. On the other hand, the exact drying temperature and technique are still debated among researchers [8,61,62]. However, the test investigation conducted by Zhutovsky et al. in 2019 [23] considering the oven drying of specimens at 60 °C until they gained constant weight was found to be more appropriate with the assumption that rigorous drying could result in a better correlation with transport properties in concrete materials. Therefore, in the present research, all the test specimens were oven dried at 60 °C for two days with the assumption of obtaining completely dried specimens with preserved microstructures before conducting the water absorption test. During preparations, each specimen was passed through a systematic procedure under controlled conditions that included the following steps:

- All surfaces of the specimen were sealed by applying silicone except on one longer side/edge (to allow water absorption) and the top face (to be used for the DICM); masking tape was applied to avoid traces of silicone, while its application on other sides was used to seal them against water absorption.
- The masking tape was only cut at designated positions on the planner face at the marked points to attach the strain gauges. It also served as a guide for the positioning of gauges in a particular direction at intended points, as illustrated in Figure 1. The face for the DICM was divided into two halves, i.e., the painted side (P) and the unpainted side (NP). On this face, eight (08) general-purpose surface strain gauges with a gauge length of 10 mm were attached. Moreover, the arrangement of gauges was kept identical on both halves in the *x*- and *y*-*axes* with respect to the direction of water absorption.

c. After the attachment of surface strain gauges, the specimen was placed in a glass chamber and kept there for approximately 24 h. Afterwards, the half face was coated with matte-type white spray paint to decrease the influence of color change and to achieve good-quality speckles for the DICM, and the other half face was left unpainted to differentiate between DICM measurements during the water absorption process. It is worth mentioning that matte-type white spray paint was used for coating because its layer did not make any thin film over the surface, and also, it does not affect the deformation measurements in the DICM (for details, readers may consult [63,64]).
d. At the end, the random speckles for the DICM were applied using red and black matte-type spray paints on the full face (*xy*-plane, as shown in Figure 2, of each specimen by adopting the procedure explained in the previous research of the authors [65]).

### 2.3. Test Methods

#### 2.3.1. Water Absorption Test (WA)

**Figure 3:**
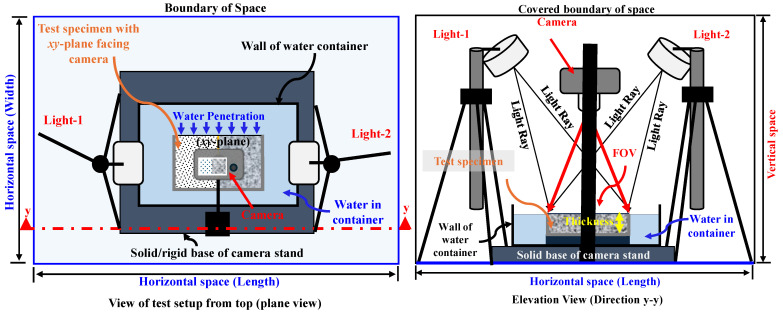
Setup for one-dimensional water test and DICM adopted in this study.

In this study, a one-dimensional water absorption test was performed on ~50 mm thick mortar specimens by adopting a test configuration allowing horizontal water absorption into concrete materials, where the absorption rate was independent of gravitational effects; rather, it was governed by hydrostatic forces and the unsaturated capillary flow in porous media of the concrete materials [14]. Indeed, a constant water depth was always maintained with specimen thickness throughout the test, as indicated in the elevation of Figure 3. In the authors’ preliminary investigations, it was confirmed that strain developments at position-1 in specimens of all strength cases stabilized after 3000 min of water absorption. Moreover, ASTM C 1585 [22] suggests water absorption for eight days (i.e., 9600 min) to characterize behaviors in concrete materials. Therefore, based on these observations/considerations and to obtain maximum evidence of changes in concrete materials, the water absorption test in this study was conducted for 160 h (i.e., 9600 min) across all specimens. Two kinds of data (i.e., recording of surface strains and capturing pictures from the top face) were acquired during the whole water absorption test/process.

#### 2.3.2. Description of Digital Image Correlation Method (DICM)

The precision and reliability of the measurements in the DICM strongly depend on the imaging system setup (e.g., light source and fixing of the camera with respect to the specimen’s flattened surface, the high-contrast surface (stochastic) patterns on specimens’ surfaces, the correlation software, etc. [34,44,54,56,66,67,68,69]). By considering all these factors, in this study, a specialized test setup was established within a disturbance-free cabin, as outlined in Figure 3. In this setup (shown in Figure 3), one high-grade charged-coupled device (CCD) camera (with a graphic resolution of 24.16 megapixels and a lens of focal distance varying from 18 mm to 55 mm) was used together with one pair of LED lights as a continuous source of lighting. This system allowed images to be captured in RAW format under a manual mode of camera, while keeping all necessary settings in the camera fixed as ISO, 200; focal aperture, 1/5.6; and shutter speed, 1/60 s (selected based on several preliminary trials). Moreover, the camera was mounted perpendicularly at ~340 mm, focusing on the top of the flat surface of the specimen (i.e., the *xy*-plane, being its field of view: FOV). It shall be noted that all the specimens in this study were of almost the same sizes, which gave an advantage as it kept the setup arrangements fixed and consistent for all specimens. However, the camera position was required to move a little up or down depending on the need to capture high-quality images. The test specimen (after preparation) was first placed inside the empty container rested over a rigid/stable camera stand (as illustrated in Figure 3) and water was poured after making all arrangements for data acquisition during the water absorption process. The camera settings and light arrangements were kept consistent for all the test series and the test was performed under controlled environmental conditions where relative humidity varied between 65.5% and 75% during testing. So, in general, the accuracy of computations in the DICM mostly depended on the quality of the applied white paint and the high-contrast speckle patterns, which were applied manually (by following the procedure described in Section 2.2.—part d).

Moreover, the strain data by surface strain gauges (as shown in Figure 1) and the digital images were continuously recorded (at one-minute intervals) from the top surface (i.e., *xy*-plane/face for the DICM) of each specimen, throughout the water absorption process. After completing the water absorption process on each specimen, the images were cropped in Capture NX-D software and imported into DIC analysis software (e.g., GOM Correlate 2019). The picture at 0 min (taken within a few seconds after pouring water) was considered as a reference or undeformed image, while considering all others as deformed images. A region of interest over the full DIC face (covering NP and P sides) was defined to analyze the state of strains after various wetting durations with respect to the reference image. This system allowed non-contact and full-field strain measurement on the full DICM face of the specimen undergoing water absorption test.

#### 2.3.3. Uniaxial Compression Test (CT)

**Figure 4:**
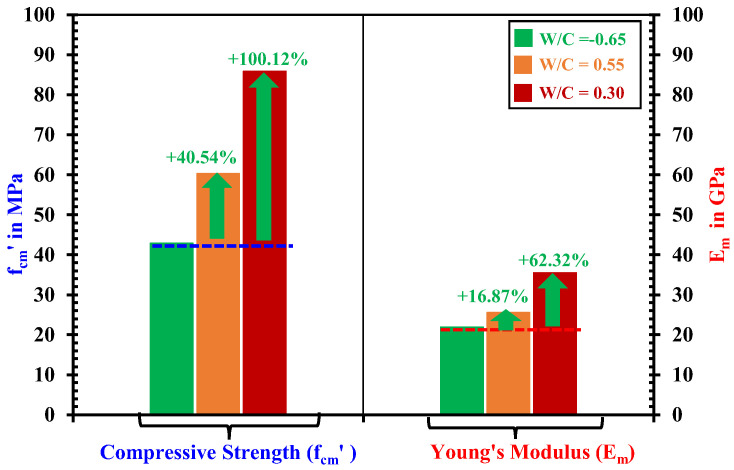
Comparison of mechanical properties between low—(*W*/*C*= 0.65), medium—(*W*/*C* = 0.55) and high—(*W*/*C*= 0.30) strength mortars determined from uniaxial compression test.

All the mortar specimens were tested under uniaxial compression along their longer length (L) after completing one-dimensional water absorption tests on them. In order to determine the deformations in the mortar specimens during the compression test, two strain gauges were attached to the left and right sides of the specimen at their middle length, and their average strain was used in stress–strain plots for each specimen. The Young’s modulus of each specimen was then determined as the initial slope on the stress–strain curve below 30% of peak stress of each specimen. Compression test results of all specimens are presented in Table 2, whereas Figure 4 displays a comparison of average values in two specimens (of each category). It is observed that in Table 2, the difference between curing and testing ages is very small; rather, both the specimens in each strength category depicted consistent results. Figure 4 shows that the medium-strength mortars exhibit 40.54% and 16.87% and high-strength mortars exhibit 100.12% and 62.32% higher compressive strength and Young’s modulus, respectively, than the low-strength mortars.

## 3. Determination of Strain Development During Water Absorption Using Surface Strain Gauges

### 3.1. Strain Development Along x-Axis and y-Axis Directions

**Figure 5:**
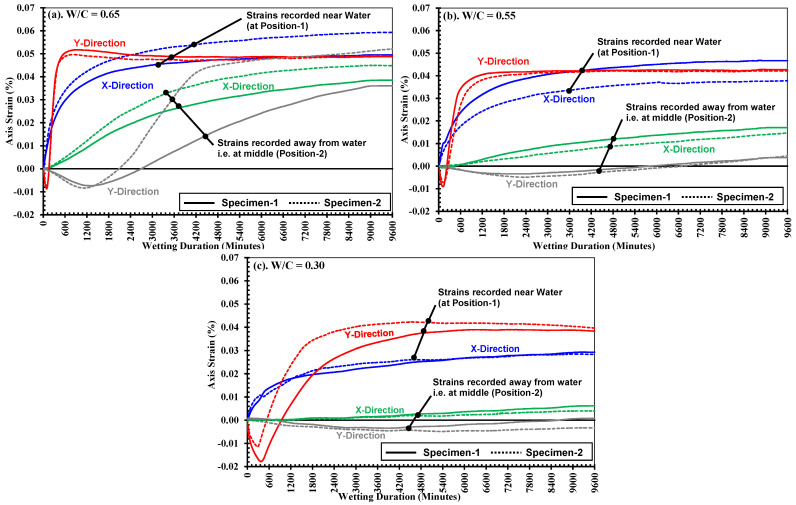
Development of surface strains measured as an average of two gauges applied on P and NP sides at position-1 and -2 in respective directions, i.e., *x*/*y axis* in strength mortars; (**a**) low: *W*/*C* = 0.65, (**b**) medium: *W*/*C* = 0.55, and (**c**) high: *W*/*C* = 0.30.

Figure 5 shows the measurements of strains on the surface of the specimens (in the *xy*-*plane*) recorded by surface strain gauges in specimen-1 (solid line) and specimen-2 (dashed lines) during water absorption in relation to the wetting duration (t in minutes) for three strength cases. In these figures, the blue and red lines show the average values of strains recorded by gauges on the P and NP sides at position-1 (near the surface in contact with water, ref. Figure 1) in the *x*-*axis* and *y*-*axis* directions, respectively. Similarly, the green and gray lines show the average values of strains recorded by gauges on the P and NP sides at position-2 (at the middle of the width of the specimen, ref. Figure 1) in the *x*-*axis* and *y*-*axis* directions, respectively. Note that the strains in the *x*-*axis* and *y*-*axis* directions indicate deformation caused by water absorption in the directions perpendicular and parallel to the water absorption direction, respectively. It is observed that the surface strain gauges in both the *x*/*y*-*axis* directions at position-1 started indicating deformations at the surfaces of the specimens immediately after contact with water, but those at position-2 were delayed in all strength cases, as observed in Figure 5 and Table 3 and Table 4. This phenomenon corresponds to the fact that water ingress has an instant effect on the volume changes in concrete materials owing to the hygroscopic nature of the cement hydrates (i.e., the C-S-H gel), which results in expansion, as illustrated in studies like [25,29,52]. Further, the volume changes observed on the small-size rectangular specimens in this study are consistent with the findings of past research conducted by Alderete et al. 2019 [25].

Moreover, Figure 5 and Table 3 clearly indicate that the *x*-*axis* direction strain in all cases has a gradual rise with the wetting duration, which in fact corresponds to the progressive increase in the level of saturation (and resulted expansion), indicating the water ingress rate within the mortars. Table 3 compares the variations in *x*-*axis* strains recorded by gauges (average of gauge on P and NP side) at positions 1 and 2, in all mortar specimens (individually). It is observed that strains, in each specimen of all strengths, consistently increased at both the positions from their lowest values at 360 min and highest at 9600 min of wetting. Also, at all-time intervals and both positions, these strains constantly decreased from across the different strengths (from low to high). For example, the M-0.65-1 of low-strength mortars exhibited a strain increase from 0.0233% at 360 min to 0.0495% at 9600 min at position-1. Similarly, for M-0.55-1 of medium-strength mortars, these strains varied between 0.0180% and 0.0467% at position-1 at 360 min and 9600 min, respectively. During the same period, strains at position-2 increased from −0.0002% to 0.0171%. And M-0.30-1 of high-strength mortars experienced the lowest strains of 0.0095% and the highest of 0.0293% at position-1 between 360 min and 9600 min of water absorption.

Moreover, upon comparing the average strains of two specimens in each case (as plotted in Figure 5), the maximum expansion strains along the *x*-*axis* (at positions-1 and 2) corresponded well to the strength of mortars; for example, the low-strength mortar experienced a maximum strain (average of two specimens) of 0.054% after 9600 min of water absorption, being the largest of the other two strengths, medium and high, which experienced maximum (average of two specimens) expansion strains of 0.043% and 0.029%, respectively, along their *x*-*axis* after 9600 min. Similarly, in the same direction, i.e., *x*-*axis* at position-2, the maximum strains (average of two specimens) recorded after 9600 min in low-, medium-, and high-strength mortars, were 0.042%, 0.016%, and 0.005%, respectively.

Table 4 compares the variations in *y*-*axis* strains recorded by gauges (average of gauge on P and NP side) at positions 1 and 2 in all mortar specimens (individually). As discussed for *x*-*axis* strains, each specimen from all strengths consistently showed increases at both the positions from their lowest values at 360 min and highest at 9600 min of wetting. It is observed in Table 4 that at all-time intervals and at both the positions, these strains constantly decreased from across the different strengths (from low to high). For example, M-0.65-1 of the low-strength mortars exhibited a strain increase from 0.0423% at 360 min to 0.0488% at 9600 min at position-1. Similarly, for M-0.55-1 of medium-strength mortars, strains at position-1 varied between 0.0175% at 360 min and 0.0427% at 9600 min, while at position-2, strains increased from −0.0011% to 0.0038% over the same period. M-0.30-1 of high-strength mortars, on the other hand, experienced the lowest strains, with values at position-1 ranging from −0.018% at 360 min to 0.0383% at 9600 min during the water absorption process.

Moreover, as shown in Figure 5, all the specimens across all strengths initially experienced compressive strains in their *y*-*axis* at both positions. These compressive strains reached maximum values (average of two specimens and two gauges at each position) of −0.0087%, −0.0090%, and −0.0179% in low-, medium-, and high-strength mortars, respectively, and similarly, at position-2 in the same direction, low, medium, and high-strength mortars also experienced strains (average strains of two specimens) of −0.008%, −0.004%, and −0.004%, respectively. These strains later changed to tensile strains at a higher rate, and later, their progression slowed down (especially at position-1), but at position-2, they continued to increase with wetting durations, as can be observed from Figure 5 and Table 4. The occurrence of compressive strains along the *y*-axis is commonly observed in all cases, which can be attributed to the bending deformation in the third axis (i.e., vertical) direction caused as part of isotropic three-dimensional volumetric expansion resulting from the internal swelling reaction of cement hydrates owing to the saturation [25,70]. Detailed behavior is discussed comprehensively in the succeeding section as a mechanism of strain development.

In general, low-strength mortars exhibited higher strains compared to medium- and high-strength mortars, whilst the high-strength mortars displayed the lowest strains overall. This indicates that as the mortar strength increases, the material’s resistance to deformation under wetting conditions also improves, resulting in progressively lower strain values.

### 3.2. Water Absorption Depth Determined by Grayscale

**Figure 6:**
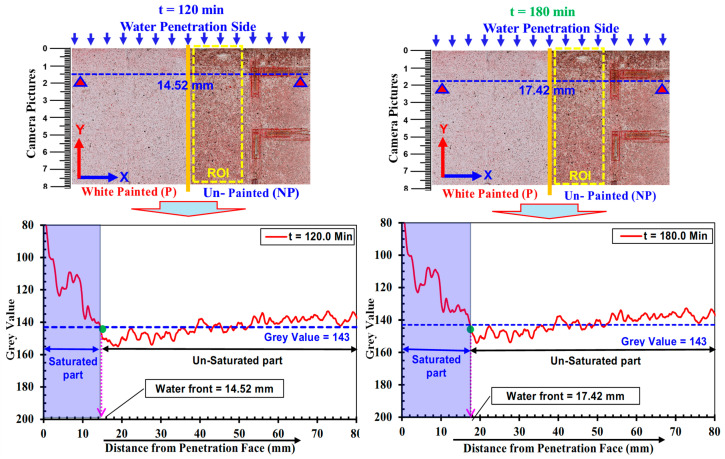

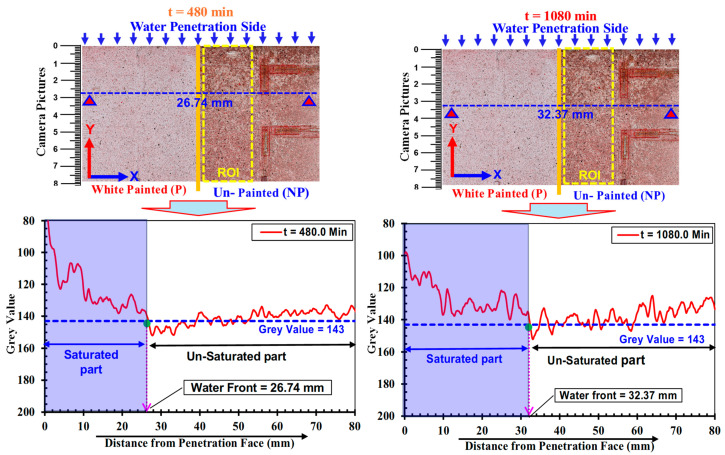
Variations in grayscale along the width of the specimen differentiating the saturated and unsaturated parts on the unpainted (NP) side in low-strength mortars (W/C = 0.65) after 120 min, 180 min, 480 and 1080 min of water absorption.

During the one-dimensional water absorption test, it was observed that the surface color was changed in the saturated area of water in the unpainted part. This observation implies that the water absorption process will be determined easily and briefly by catching the border of the color change part. In this image data, each pixel worked as an independent detector and was assigned a gray level according to the surface contrast/color during the water absorption process, i.e., the saturated parts have lower gray values compared to the unsaturated part owing to the darkened texture on the unpainted (NP) side part of the specimen surface, as indicated in past studies, e.g., [17,71]. The images taken during wetting, at different time intervals, were imported into ImageJ (Fiji) software, where the gray levels were converted to 8-bit data. Figure 6 shows the original pictures (upper row) taken after 120, 180, 480, and 1080 min of the water absorption process in low-strength mortars, with dashed yellow lines indicating the region of interest (ROI). To better visualize the variations in grayscale values in the saturated region, the averaged grayscale values were plotted along the *y*-*axis* (solid red line in lower graphs) with the water absorption side being at 0 mm. It is necessary to note that these grayscale values were averaged within smaller dimensions of ROI, which varied along the *y*-*axis* depending on image contrast changes due to saturation by water. A clear difference in the grayscale values in the saturated and unsaturated regions was observed, i.e., varying gray values in the saturated part were noticed, whereas in the unsaturated part, it seemed almost constant. The gray value distribution profiles formed a triangular shape within the saturated regions.

The water absorption depth (waterfront) was estimated from these profiles (Figure 6) by identifying the point where the red curve (representing the gray value at a specific point along the *y*-*axis*) intersected the horizontal blue line (a threshold grayscale value) The threshold value was defined at 143 by using the maximum gray value of captured images from the surface of the specimen (i.e., low strength) in its unsaturated regions. It was discovered that the triangular region expanded with increasing wetting durations, indicating the progressive movement of the waterfront and increased saturation over time.

### 3.3. Three-Dimensional Deformation Behavior During Water Absorption

**Figure 7:**
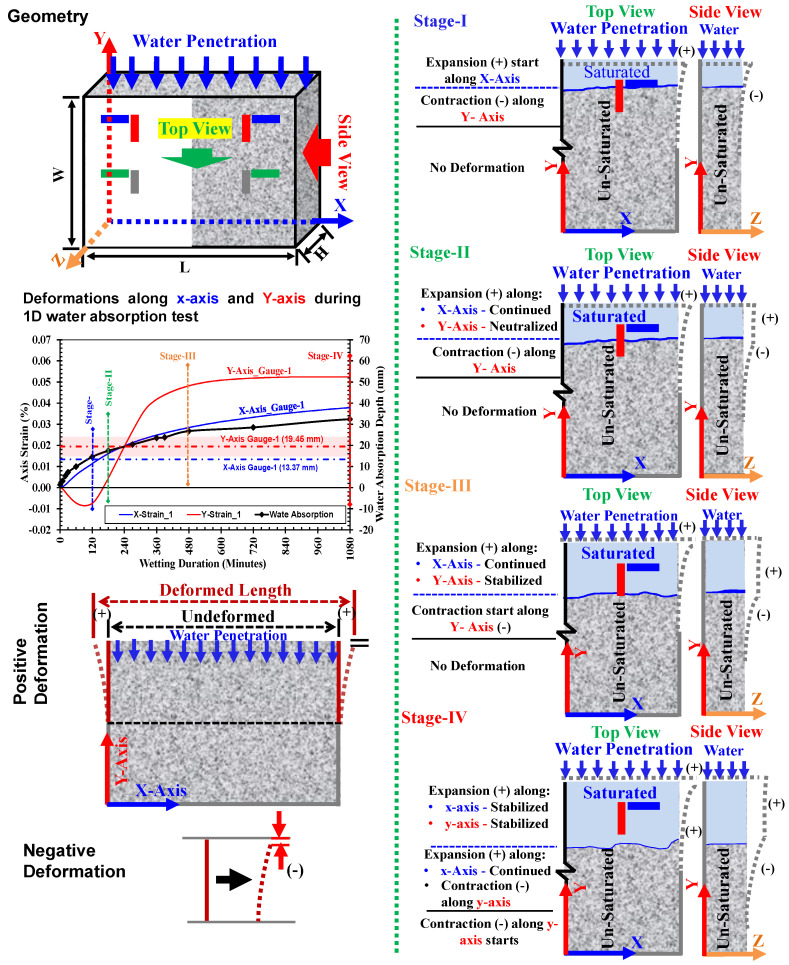
Schematic representation of volumetric changes resulted from water absorption in mortar specimens.

Figure 7 presents the schematic illustration of the possible volumetric changes occurring in the mortar samples during the first 1080 min of the water absorption test in order to discuss strain development. In this figure, the isometric deformation states of the mortar specimens are considered at stages I, II, III, and IV after 120, 180, 480, and 1080 min after starting the water absorption test, respectively. The axis strain versus wetting duration plot represents the *x*-*axis* and *y*-*axis* strains recorded by surface strain gauges installed at position-1 in the low-quality specimen. Additionally, the water absorption depth determined on the NP side of the same specimen by considering changes in gray values is plotted. As highlighted in Section 3.1, the mortar specimen undergoes isotropic three-dimensional volumetric expansion resulting from the internal swelling reaction of cement hydrates. In these figures, dashed gray lines indicate the magnified deformed shape of the sample from the top view and side view (ref. geometry). The blue lines in figures showing top and side views indicate the waterfront at a particular stage (judged based on changes in gray values at the surface on the NP side, ref. Figure 5).

Observations made at each stage are explained below as follows:

- At stage-I: The waterfront reached the *y*-*axis* gauge (red mark) at position-1, as shown by the blue line in the top view, indicating the possible expansion along the *x, y*, and *z*-axis of the specimen in its saturated part. Since the portion below the gauge was still unsaturated, this part exhibited contraction due to small bending occurring in the *z*-*axis* direction. However, the positive strain in the *x*-*axis* was observed owing to the uniform expansion.
- At stage-II: The half of the *y*-*axis* strain gauge was covered in the saturated part (indicating positive strain) and half unsaturated (negative strain occurs due to contraction as a result of bending deformation). It is true, therefore, that both the strains balanced each other, and the *y*-*axis* strain became neutralized. But at the same time, the *x*-*axis* strain continued to show a gradual increase due to continued expansion.
- At stage-III: The moment when the full underneath part of the *y*-*axis* gauge was saturated and there was the maximum possible deformations along the *y*-*axis* was reached. There was a rapid increase in the *y*-*axis* strain just before reaching this stage, e.g., when water progressed through its gauge. But soon after the full part below the *y*-*axis* strain gauge became saturated, and the stabilized condition in expansion was achieved.
- At stage-IV: The water absorption increased, resulting in more progressed volumetric changes in the specimen.

## 4. Implementation of DICM to Evaluate Strain Development During Water Absorption

### 4.1. Spatial Distribution of Strain During Water Absorption in Low-Strength Mortars

#### 4.1.1. Variation in Strain Along *x*-*Axis*

**Figure 8:**
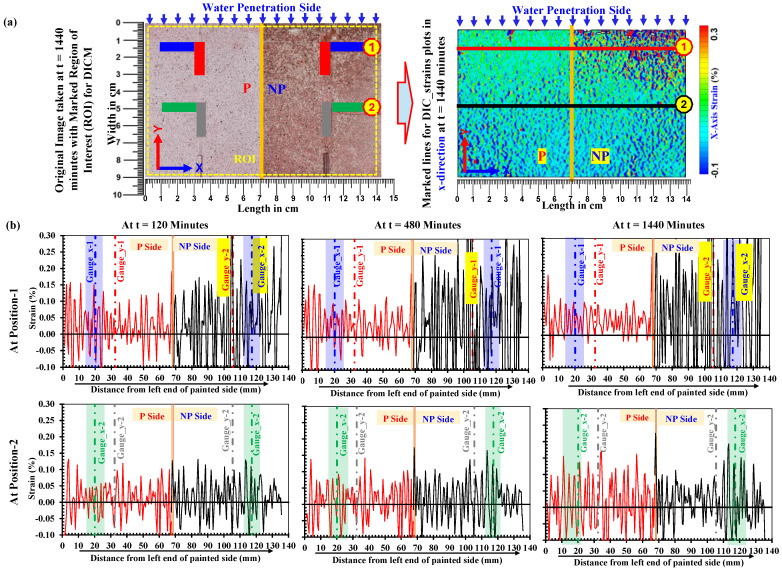
Plots of *x*-*axis* strain variations in low-strength mortars (W/C: 0.65) on painted and un-painted sides at position-1 and -2 with increasing wetting durations. (**a**) **Left:** Marked region of interest for DICM on image taken after 1440 min; **right:** illustration of reference lines along *x*-*axis* at which strains are plotted in DICM, (**b**) *x*-*axis* strain distribution in DICM along line-1 and -2 after 120 min, 480 min and 1440 min of water absorption.

In Figure 8, the variations in the *x*-*axis* strains on the painted (P) and unpainted (NP) sides of the low-strength mortar specimen obtained by DICM are given at t = 120, 480, and 1440 min. The results of low-strength mortar were selected to validate the DICM because the water absorption and strain development are emphasized rather than other cases. The two horizontal section lines are created at gauge positions-1 (red) and -2 (black), as indicated in the full field strain map of the *x*-*axis* strain considered at t = 1440 min in order to check the variations in *x*-*axis* strain measurements in the DICM. The strain distribution along the full length of these lines is given in Figure 8a. It is pertinent to mention that these plots are considered without any treatment (post-processing like smoothening, etc.), enabling a realistic overview/judgment over the strain calculations in the software. The large variations in strains by the DICM are observed in the NP region of the specimens. It is notable that these large variations are observed mainly at position-1 as a result of image contrast change (clearly observed on NP side) because of the increased degree of saturation with an increased wetting duration, but at position-2, similar strain tendencies in grayscale values are observed on both the P and NP sides, which are clearly due to no change in the image contrast because water did not reach there.

Thus, in the DICM, an unpainted surface shows high strain intensity with large variation in water-saturated regions compared to the painted side surface; the strain gauges, however, depict almost similar responses on both sides. Figure 6 also reveals that the surface image contrast is changed in the saturated part on the NP side, and there would be high chances of complete changes in distinct patterns in the target image, which can lead to errors in strain calculations in the DICM. This is because, in the DICM, strain is computed from changes in displacements while tracking grayscale of the distinct patterns within a subset, after establishing certain correlation/matching criteria between subset pixels in the reference image and the target image for accurate calculations [34,55,66,67,68,72,73]. Grayscale is clearly changing between reference and target images in the saturated part on NP side. Therefore, the strains on the NP side most likely include computational errors contributed from the established correlation criteria; therefore, these are considered unrealistic/pseudo strains and can be regarded as unnatural.

#### 4.1.2. Variation in Strain Along *y*-*Axis*

**Figure 9:**
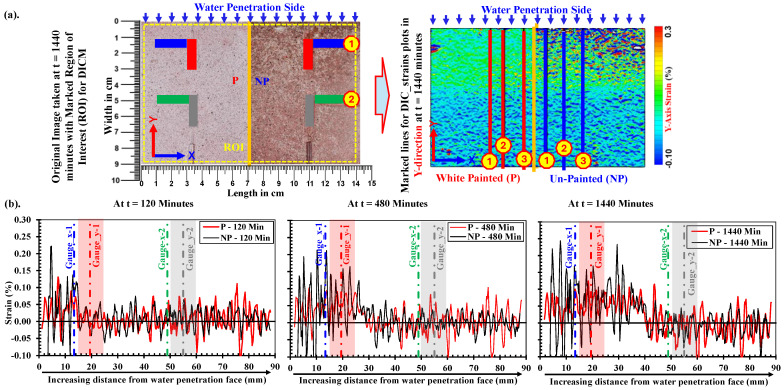
Plots of *y*-*axis* strain variations in low-strength mortars (W/C: 0.65) on paint and no paint side at position-1 and position-2 with increasing wetting durations. (**a**) Illustration of reference lines along *y*-*axis* at which *y*-*axis* strains are plotted, (**b**) *y*-*axis* strain distribution in DICM along *y*-*axis* at P and NP side after 120 min, 480 min and 1440 min of water absorption.

Figure 9 shows the variations in the *y*-*axis* strains on the painted (P) and unpainted (NP) sides of the low-strength mortar specimen at t = 120, 480, and 1440 min in the low-strength mortar specimen. These variations are taken as an average of three lines on each side (P and NP), as indicated in the full field strain map of *y*-*axis* strains at t = 1440 min. The strain distribution along the full length of these lines (i.e., the width of the specimen) is reproduced in Figure 9, where the black line shows an average strain along three lines on the NP side and the red line shows an average strain along three lines on the P side. In a similar manner as for *x*-*axis* strains in Section 4.1.1, these plots are considered without any treatment of data from DIC software (e.g., GOM Correlate 2019 v2.0.1). Moreover, Figure 9 highlights the strain distributions along the *y*-axis on the NP side, which also exhibited large variations in data compared to those on the P side at the same time interval in the saturated part.

### 4.2. Strain Recorded by Surface Strain Gauges vs. Strains by DICM (Pε__DICM_ vs. Pε__Gauges_)

**Figure 10:**
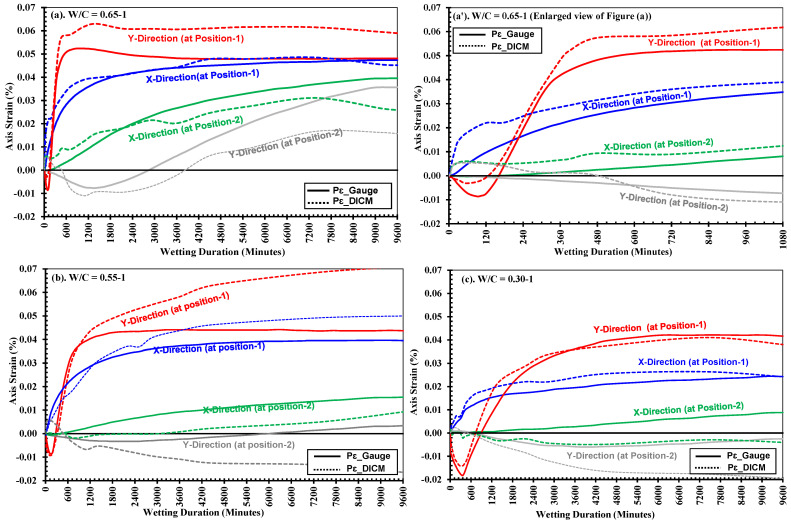
Comparison of strains calculated in the DICM with the strains recorded by surface strain gauges on the P side of specimens during water absorption in (**a**) low: W/C = 0.65; (**b**) medium: W/C = 0.55 and (**c**) high: W/C = 0.30, strength mortars. Where plot (a’) in Figure 10 indicates the enlarged view of plot (a) at initial stage in low strength mortars.

Figure 10 shows the measurements of strains on the surface of the mortar specimens (in the *xy*-*plane*) recorded by surface strain gauges (solid line) and their comparison with the strains calculated in the DICM (dashed lines) with increasing wetting durations. Additionally, Table 5 compares the strains (*x*-*axis*) by the DICM across all specimens in different strength cases at various time intervals (i.e., 360,1440, 2880 and 9600 min). Moreover, the strain results on the painted side gauges are considered for comparison in all strength cases. Generally, the results obtained from correlation algorithms, in the DICM, cannot directly be used owing to the noise in the received data, which may have either inherently generated due to the limitations in the equipment, the used algorithms or the data processing parameters (e.g., subset size, subset shape function, etc.) [34,66,72]. To address such issues, some techniques or algorithms are recommended to recognize, describe and remove the noise from such data without altering their actual character to interpret it. An efficient choice to achieve reasonable accuracy involves optimal smoothing of the data centered over a specific region [73]. Therefore, in this study, centrally oriented (mean) [74] strain variations in the spatial domains of two neighboring points on either side were determined. Moreover, the strains by the DICM shown in Figure 10 were obtained as an average of strain values within the length equal to that of the gauge (i.e., 10 mm) over the section lines drawn parallel to the installed surface strain gauges. Figure 10a’ elaborates the variations in strains on an enlarged scale for low-strength mortars. These values are also compared in Table 5 for different wetting durations ranging from 360 to 9600 min. It can be observed from Figure 10 and Table 5 that calculations on the painted side using the DICM consistently align with gauge strains (with minor variations) across low-, medium- and high-strength mortars as wetting durations increase. For instance, in M-0.65-1, the DICM recorded a strain of 0.0286% after 360 min, compared to a gauge strain of 0.0217%, showing a 0.0069 difference in absolute values. At 9600 min, the DICM measured 0.0452%, while the gauge strain was 0.0474%, resulting in a 0.0022 difference in absolute values. Similar trends were observed across all strength classes at various wetting durations.

Thus, strains calculated in the DICM were found to be in good agreement with those measured by surface strain gauges. This confirmed the verification of the implementation of the DICM during the water absorption test. It was further observed that the results included slight out-of-plane displacement caused by the bending effect owing to three-dimensional isotropic deformations during the water absorption. However, it was expected that the effect was negligible owing to the very small size of the test specimen; moreover, the out-of-plane displacement occurred only for the initial times, and thus, overall behavior can be evaluated successfully by applying the DICM.

### 4.3. Evaluating Water Absorption Characteristics Obtained by DICM

#### 4.3.1. Full Field Deformations in Mortars Along *y*-*Axis* Direction

**Figure 11:**
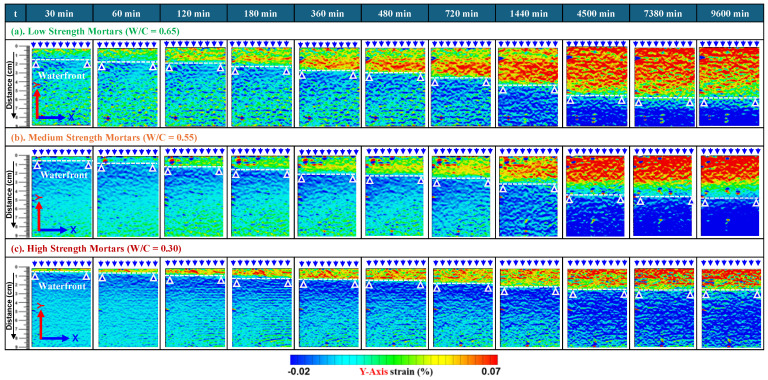
Comparison of full field strain distribution along y-axis with increasing wetting durations with reference to state just before starting water absorption in specimen-1 of different-strength mortars, i.e., (**a**) low strength: *W*/*C* = 0.65; (**b**) medium strength: *W*/*C* = 0.55; and (**c**) high strength: *W*/*C* = 0.30.

Figure 11 presents the strain distributions along the *y*-*axis* on the DIC face on the paint side of low-, medium-, and high-strength mortar specimens obtained after DIC analysis by using digital images captured during the water absorption process. As explained in Section 2.3.2, a picture, taken after a few seconds of contact with water, was considered as a reference/undeformed image (i.e., t = 0 min). And those taken at later times during water absorption were considered deformed images due to water absorption in their saturated regions. The mechanism explained in Figure 7 can also be understood through these strain maps. Additionally, these strain distributions show that areas with positive strain intensities progressively increase as wetting durations increase in the direction of water penetration/absorption, and the swelling of hydrates is directly correlated with longer saturation times. Moreover, the marked white horizontal lines on the strain maps indicate the position of the waterfront and the progression of water absorption with the increased time of the continued water supply. Interestingly, through these maps, it was observed that, at the same time, these maps not only indicate the progression of water during the absorption process but also the level of deformations experienced by different mortars depending on their pore fractions and help to clearly distinguish between different strength/quality states of concrete materials.

#### 4.3.2. Comparison of Progression of Full Field Deformations in *y*-*Axis* Direction

**Figure 12:**
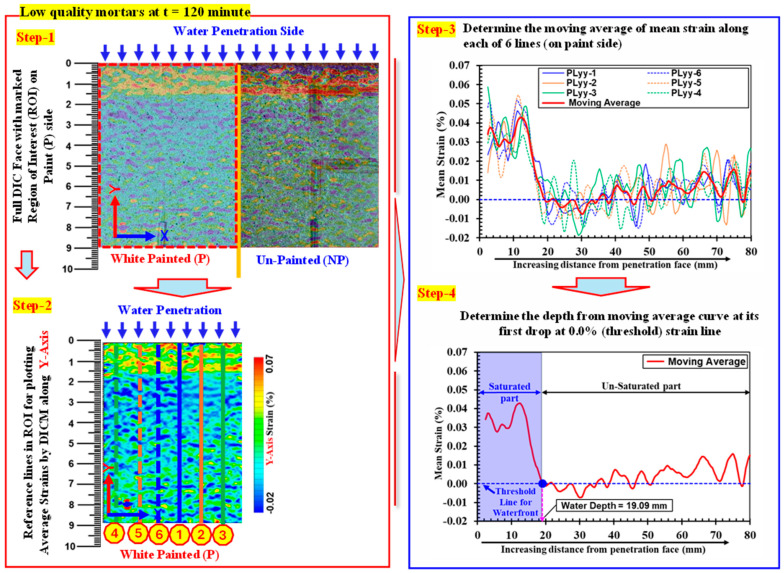
Definition of determining water absorption depth by employing strain distribution along the *y*-*axis* using DICM.

From strain distributions along the *x*- and *y*-*axis* recorded by gauges (Figure 5 and Figure 10), it was observed that the progression of the *y*-axis strain is prominent during one-dimensional water absorption because the water movement is also predominantly along the *y*-*axis*. Therefore, the water absorption depth in this study is determined by considering strain plots along the *y*-*axis*. Figure 12 explains the procedure to determine the water absorption depth (as an example) in a low-strength mortar specimen at t = 120 min. In this procedure, at first, the mean value of *y*-axis strains was calculated along six equally spaced lines drawn along the width (W), i.e., the *y*-*axis* on the painted side starting at 0 mm (near water). Thereafter, one moving average line was determined from these lines to determine the water depth. As stated earlier in Section 3.1, the strain value suddenly changes from tension to a small compression value at the waterfront, and the water absorption depth is defined by this change point (i.e., 0.0% *y*-*axis* strain) in all specimens of this study.

**Figure 13:**
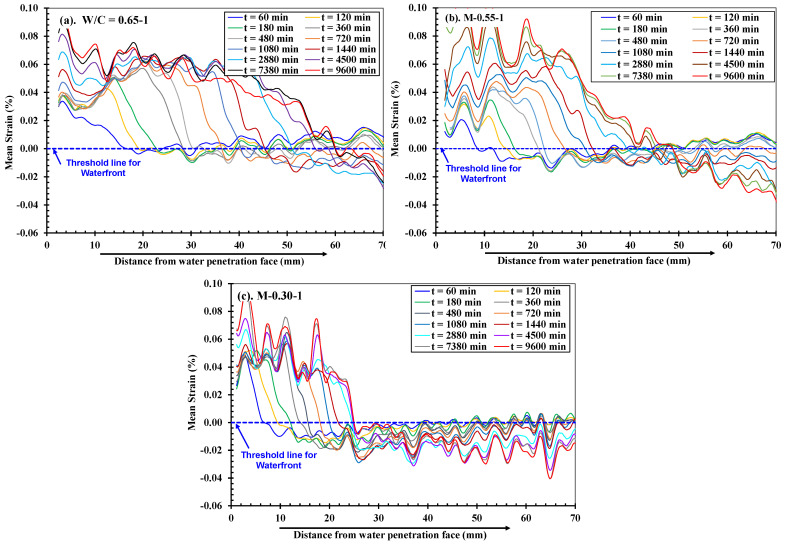
Distribution of strain by the DICM along *y*-*axis* on the P side of the specimens in (**a**) low: W/C = 0.65; (**b**) medium: W/C = 0.55; and (**c**) high, W/C = 0.30 strength mortars. **Note:** Points on each line for a specific time in these graphs crossing threshold strain corresponds to water absorption depth.

Figure 13 shows the mean value of the *y*-*axis* strain (in all six specimens) plotted as a moving average along the *y*-*axis* at different time intervals considered with respect to the specimen’s initial contact with water. As the wetting duration increased, a shift to the right in the strain distribution curves in all cases was observed, which confirmed the progressive increase in the swelling of cement hydrates and hence the increased water absorption. It was further observed that the lower the strength was, the higher the tendency of shifting towards the right was, signifying the higher water absorption rate. All the specimens experienced negative (compressive) strains in their unsaturated parts owing to the degree of saturation achieved in the saturated part. It was also noticed that in the low-strength mortars, the tendency toward the shift in these strain curves was higher, and the absorption was higher, indicating more swelling in the saturated part, and consequently, more compressive strain in the farther points from the water was observed as compared to the medium- and high-strength cases.

**Figure 14:**
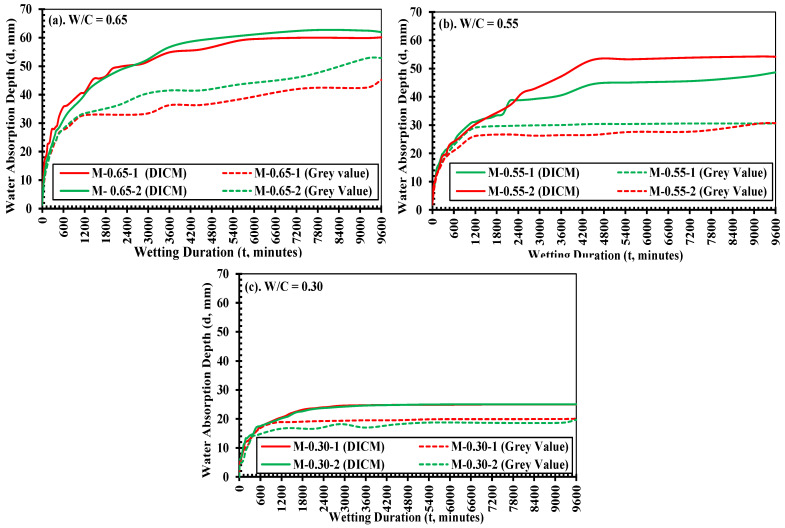
Comparison of water absorption depths with increasing wetting durations determined by DICM and by variations in grayscale in specimen-1 and -2; (**a**) low: *W/C* = 0.65; (**b**) medium: *W/C* = 0.55; and (**c**) high, *W/C* = 0.30 strength mortars.

The water absorption depths determined by observations on variations in grayscale in the unpainted part and the strain distributions in the DICM in the painted part are plotted in Figure 14 as a function of wetting duration (i.e., 9600 min was considered in this study, the same as that suggested for sorption tests according to ASTM C 1585 [22]). In this figure, solid and dashed lines show the results of the water absorption depths determined by observations in the DICM and grayscale in specimen-1 (red lines) and specimen-2 (green line) of each strength, respectively. It is observed that apart from differences in the curing ages, both the specimens in each strength case depicted similar water absorption characteristics, either determined by grayscale variations or by the observations in the DICM. This can be attributed to the fact that after a longer curing time (76 days or higher in this study), the sufficiently densified microstructure of cement pastes was observed [1,7,10,29,75]. However, it is strongly recommended to deeply consider the curing ages while characterizing water absorption behavior in cement-based materials.

Moreover, as in the sorption test (ASTM C1585 [22]), two distinct stages of water absorption can be clearly observed: an initial rapid rate of absorption followed by a slower rate. The later stage approaches a plateau due to the increased saturation level, which reduces the connectivity of capillary pores. This reduction is attributed to the refinement of the microstructure in cement-based materials, resulting from the formation of new hydration products (e.g., C-S-H or any hygroscopic) through the rehydration of unhydrated cement paste [29,70]. These test observations adequately correlate with the findings from previous studies [2,14,23,59,76], which utilized gravimetric measurements.

It is also observed that the DICM resulted in relatively higher water absorption depths compared to those determined by grayscale imaging at the same time, using the NP side of the same image. However, this difference was small during the initial few hours. The said mechanical behavior is attributed to the fact that the DICM can capture even very small deformations at any saturation level during the absorption process, whereas grayscale imaging may not distinguish color changes in the relatively less saturated parts (which are expected at the waterfront). Another reason could be that the paint side protected the surface from immediate drying, while the NP side was directly exposed to the air, potentially affecting the moisture at the waterfront.

Results reported in Figure 14 and Table 6 show the water absorption depths at increasing wetting durations with respect to the specimen’s first contact with water. A clear difference between the absorption behaviors of the mortars with different mix compositions (i.e., W/C ratios) or their strength states is observed, e.g., water absorption depth in low-strength mortar (i.e., *W/C* = 0.65 in this study) was always significantly higher than the other two strengths owing to its more porous media than the other two strengths. More specifically, it can be observed that after 360 min (6 h), the water penetrated in the low-strength mortar to a depth over 25 mm, which progressively increased to even more than 40 mm after 1440 min (24 h). It can be understood that even within the first 24 h of contact, water crossed the limit of the minimum recommended concrete cover thickness, i.e., 38 mm for mild exposure conditions of the retaining structures ACI 318-19 [77]. It was observed that the water absorption depth continued to increase with wetting duration for about 9600 min (160 h) and eventually reached about 60 mm.

Although for the medium-strength mortar, the water absorption depth is smaller than that of low-strength mortar, which initially was about 20 mm after 360 min (6 h) of the absorption process and 40 mm after 2880 min (48 h), it crossed the reference value of cover thickness in design and eventually reached about 50 mm after 9600 min. For the high-strength mortar, the water penetrated about 20 mm after 1440 min (24 h). However, water did not penetrate to that much of an extent afterwards and eventually stayed at about 25 mm. Moreover, these displayed behaviors/trends water absorption depth are similar to the results obtained through gravimetric measurements in accordance with ASTM C1585 [22], during the water absorption process in cement-based materials, i.e., the initially rapid rate of absorption (calculated as the slope of the best-fit regression line for points on the obtained curve up to the first 6 h) and then the slow rate of absorption (calculated as the slope after 48 h), with a transition period between these two stages.

**Figure 15:**
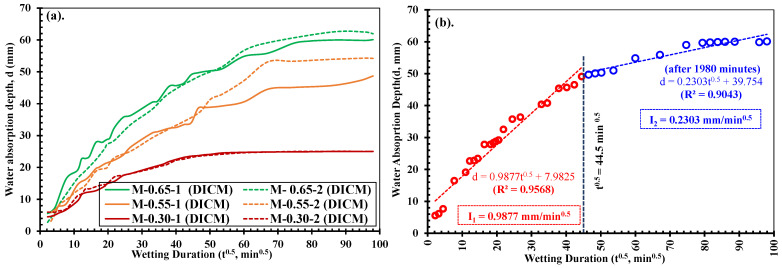
Relationship between the water absorption depth (d) determined by the DICM and the square root of wetting time in (**a**) low: W/C = 0.65; medium: W/C = 0.55; and high: W/C = 0.30 strength mortars, and (**b**) determination of absorption rates in low-strength mortar specimen-1 (I_1_: slope of best fit line up to 1980 min; I_2_: slope of best fit line after 1980 min of starting absorption).

Figure 15 illustrates the relationship between water absorption depth (d), as determined by the DICM, and the square root of time (t^0.5^) for all the test specimens. In this plot, the slope of the best-fit lines passing through all the points up to 44.5 min^0.5^ defines the rapid/primary rate of absorption (I_1_), and beyond this point, defines the slow/secondary rate of absorption (I_2_) in all strength cases. Like in Figure 15b, I_1_ and I_2_ in specimen-1 of low-strength mortar were determined as 0.988 mm/min^0.5^ and 0.230 mm/min^0.5^ before and after the deviation point (i.e., 44.5 min^0.5^) with regression coefficients (R^2^) of 0.9568 and 0.9043, respectively. Similarly, by adopting the same procedure, the absorption rates (i.e., I_1_ and I_2_) in all the test specimens were determined. It shall be noted that in M-0.55-2, the deviation point was at 60.0 min^0.5^ instead of 44.5 min^0.5^, as commonly observed for all other cases, which can be attributed to the apparent change in the strains in the DICM [34,69,72], which may have occurred during the water absorption test.

Table 7 summarizes the primary and secondary rates of absorption for all the mortar specimens determined from Figure 15 with the corresponding regression coefficients (R^2^). According to [78], R^2^ > 0.85 indicates an excellent correlation between the fitted parameters. In this study, all the specimens exhibited excellent linear correlations, particularly in the primary rate of absorption, which varied between 0.9560 and 0.9867, resulting in very close values between each specimen of a strength category. In contrast, lower correlation coefficients were observed for I_2,_ which ranged between 0.5776 and 0.9512. As an average of the two specimens in each strength category in the primary rate of absorption, the medium- and high-strength mortars exhibited 30.14% and 59.78% lower initial water absorption rates compared to the low-strength mortars. On the other hand, the secondary rates of absorption corresponded well with the absorption depth with very close values in each specimen except for M-0.55-2, which also confirms that curing of medium- and low-strength specimens after 78 days did not affect the primary absorption behavior in the mortars.

## 5. Relationship Between Water Absorption Characteristics and the Mechanical Properties of Mortars of Different Strengths

### 5.1. Relationship Between Rate of Water Absorption (I) and the Mechanical Properties

**Figure 16:**
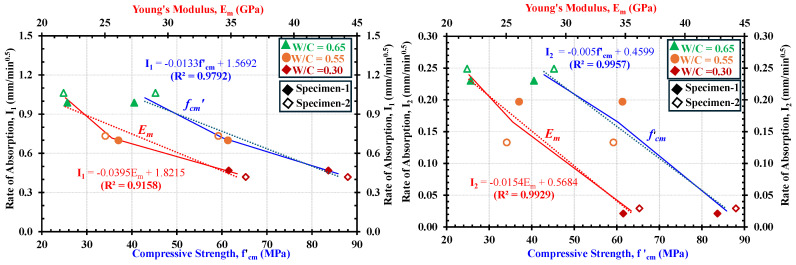
Relationships between mechanical properties (f′_cm_ and *E_m_*) of different-strength mortars with primary (I_1_) and secondary (I_2_) rates of absorptions determined in this study by observations in DICM: (**a**) (f’ _cm_ and E_m_) vs I_1_; (**b**) (f’_cm_ and E_m_) vs I_2_.

The water absorption rates (*I*_1_ and *I*_2_) in mortars of different strengths are plotted against their mechanical properties, namely, compressive strength *f′_cm_* (blue line) and Young’s modulus *E_m_* (red line), as shown in Figure 16a and b, respectively. A decreasing trend is observed in these relationships, which clearly highlights that the water absorption in mortars is strongly related to their compressive properties. That is why the specimens with higher water absorption rates (e.g., low strength) tend to have lower compressive strength and Young’s modulus. In general, the correlations of both *f′_cm_* and *E_m_* with the absorption rates depicted consistent behaviors. However, these differ between the rates of absorption, i.e., primary (Figure 16a) and secondary (Figure 16b), for the same specimens. For instance, the linear relationship of *f′_cm_* and *I_1_* in Figure 15a shows a higher decreasing slope of −0.0133 from low-strength to high-strength mortars than that in the case of *I*_2_, which was lower, at −0.005 in Figure 16b. This phenomenon can be attributed to the fact that slow absorption rate (i.e., *I*_2_) is more significant in high-strength mortars than in the low- and medium-strength ones owing to the reduced potential of capillary pores to take more water [11,13,50,79].

### 5.2. Relationship Between Maximum Developed Strain Along x-Axis (i.e., ε_xx_) in Fully Saturated Regions and the Mechanical Properties

**Figure 17:**
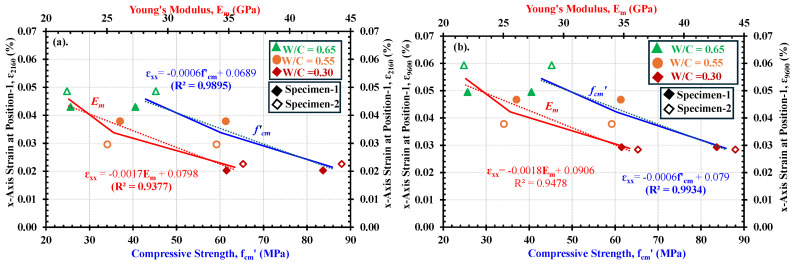
Relationship between mechanical properties (f′_cm_ and E_m_) and the strain along the x-axis in the fully saturated part: (**a**) at 2160 min (beginning of slow absorption); and (**b**) at 9600 min (absorption test stopped).

Figure 17a and b show the relationship between the maximum expansion strain (*ε_xx_*_-1_) and the mechanical properties, i.e., compressive strength, *f′_cm_*, and the Young’s modulus, *E_m_*, of mortars after 2160 and 9600 min of the water absorption process, respectively. It shall be noted that these durations were selected based on observations as illustrated in Figure 15b, i.e., 2160 min being the beginning of slow absorption and 9600 min being considered a time after which no further development in expansion at position-1 would happen. The expansion strain (*ε_xx_*_-1_) included in Figure 17a and b can be defined as the strain obtained by the DICM at position-1 of gauges. The *x*-*axis* strains in all specimens of low-, medium-, and high-strength mortars at position-1 are considered because it is deemed that at this point, the maximum possible expansion due to saturation has occurred in all strength categories. It is examined from Figure 17a,b that the deformability of the mortars during the water absorption process tends to reduce with increased compressive strength, (*f′_cm_*) and Young’s modulus (*E_m_*). Similarly to the relationship between compressive properties with rate of water absorption (shown in Figure 17), a higher linear correlation is achieved in these relations, which emphasizes a strong dependence of the strain generation on the microstructural condition of the mortars.

Table 8 compares the mechanical properties and water absorption characteristics of mortars across low-, medium-, and high-strength cases. Low-strength mortars exhibited the lowest mechanical properties, with compressive strength (*f’_cm_*) ranging between 40.52 and 45.24 MPa and Young’s modulus (*E_m_*) between 40.52 and 45.24 GPa. These values were lower than those of medium-strength mortars (*f’_cm_*: 61.33–59.21 MPa and *E_m_*: 26.08 and 25.04 GPa and significantly lower than high-strength mortars (*f’_cm_*: 83.66–87.97 MPa) and *E_m_*: 22.02–21.72 GPa). In contrast, water absorption characteristics showed opposite trends. For example; low-strength mortars depicted the highest absorption rates (*I*_1_: 0.988–1.063 mm/min^0.5^) and expansion strains (*Ɛ_xx_*_-1 (2160)_: 0.0429%–0.0486%), exceeding those of medium-strength mortars (*I*_1_: 0.699–0.733 mm/min^0.5^) and expansion strains (*Ɛ_xx_*_-1 (2160_: 0.0379%–0.0296%). But the high-strength mortars experienced the lowest absorption rates ***(****I*_1_: 0.988 and 1.063 mm/min^0.5^) and expansion strains (*Ɛ_xx_*_-1 (2160)_: 0.0203% and 0.0207%). Similar trends were observed for strains after 9600 min and I_2_.

From Figure 16 and Figure 17 and Table 8, it is understood that the low-strength mortars exhibited the lowest values in mechanical properties but the highest rates of water absorptions and expansion strains compared to medium- and high-strength mortars. Also, the observed rates of absorption and the deformability during both the rapid and slow absorption processes are directly proportional to each other across all strength classes. Therefore, understanding either of these factors could assist in estimating the likelihood of a potential deterioration process in concrete materials.

## 6. Conclusions

In this study, a one-dimensional water absorption test was conducted on rectangular mortar specimens from three different strengths (categorized as low, medium, and high), and the characteristics, including water absorption depth/rate and the strain generation at the flat surface of the mortar specimens, were evaluated by utilizing DICM. The relationships between these characteristics and the mechanical properties of the same specimen yielded good correlations. The main conclusions can be drawn as follows:

1. Water ingress has an instant effect on the volume changes (expansion) in the cement-based materials owing to the hygroscopic nature of the cement hydrates (i.e., the C-S-H). Moreover, the continuous monitoring/evaluation of strains at specimens’ surfaces during the water absorption process indirectly provides physical evidence of such an internal swelling phenomenon in concrete materials during the process of water absorption.
2. The DICM is an effective method to evaluate the volumetric changes in concrete materials by evaluating the strains at the specimen surfaces during the water absorption process. However, the calculations in the DICM are strongly influenced by the changes in the contrast of the image within the saturated regions of the test specimen surfaces and result in pseudo strains. Therefore, applying the DICM with water absorption in concrete materials requires their surface to be coated with matte-type spray paints first and then enhanced by speckling to achieve good test results.
3. The deformability and the water absorption depth/rate in concrete materials are directly related to each other and depend on their mix compositions. The progression in surface strains measured in the DICM provided more reliable evaluations of water ingress rates into the mortars, thereby indicating the closest boundary between primary and secondary liquid water absorption processes. The water absorption depths and the tendencies in expansion strains in saturated parts were different for specimens of three different strengths. However, in all three strengths, the depth (d) vs. square root time (t^0.5^) curves deviated from a linear relationship after the same time, i.e., 1980 min, resulting in highlighting that the largest strains occur within the primary absorption period, after which, strains progress slowly owing to the slow water absorption in the secondary absorption period.
4. Concrete materials with lower w/c ratios (high strength) experience lower water absorption rates. On the other hand, mortars with lower strengths will allow more rapid water absorption and other liquids or ions owing to the presence of more pore fractions. Moreover, the primary and secondary absorption rates showed almost linear relationships with mechanical properties such as compressive strength and the Young’s modulus of mortars.
5. The volume changes in mortars due to water absorption in the saturated part have a clear relationship with the mechanical properties. The results indicated that the evaluated durability properties related to water permeation not only relate to the water absorption rate but also to the compressive strength, Young’s modulus, and the volume change due to saturation.

In this study, the DICM was employed to determine the water absorption characteristics in ordinary mortar specimens with simply set test arrangements. The test results yielded excellent relationships between mechanical properties and water absorption characteristics, which are good indicators for assessment of the durability in concrete materials. However, some limitations incurred, like out-of-plane bending, can affect the accuracy of the measurements. In the future, the established framework will be extended in various aspects by improving the precision in tackling out-of-plane bending, and it will be involved in the evaluation of absorption characteristics in slices cut from core samples of an existing structure, checking the durability of concrete at high temperature, materials designed/proposed for sustainable constructions and so on.

## Figures and Tables

**Table 1:** Mix proportions of the test specimens correspond to various target strengths.

Strength Category	W/C	Contents Unit Weights (kg/m^3^)				
		W	C	S	AE	SSP-104
Low	0.65	295	454	1135	5.35	-
Medium	0.55	295	535	1339	5.35	-
High	0.30	165	555	709	-	3.05

W: water, C: cement, S: sand, AE: air entrainment agent, SSP: superplasticizer.

**Table 2:** Details of test specimens and their mechanical properties.

Strength	Designation ^1^	Dimensions in mm (L × W × H)	Age (Days)			Mechanical Properties ^2^	
			Curing	WA	CT	f_cm_’ (MPa)	E_m_ (GPa)
Low	M-0.65-1 M-0.65-2	152.5 × 100.8 × 50.3 151.6 × 101.7 × 50.2	76 145	89 157	124 174	40.52 45.24	22.02 21.72
Medium	M-0.55-1 M-0.55-2	151.4 × 102.4 × 50.2 152.6 × 102.1 × 50.6	78 145	102 157	124 174	61.33 59.21	26.08 25.04
High	M-0.30-1 M-0.30-2	153.3 × 100.5 × 50.4 151.3 × 100.2 × 50.0	32 32	42 49	56 84	83.66 87.97	34.82 36.18

WA: water absorption, CT: Compression Testing, f_cm_’: compressive strength of mortars, E_m_: Young’s modulus of mortars; ^1^ Designations will be used in plots, M (mortar) – W/C ratio – specimen number. ^2^ Properties will be used for correlation with water absorption characteristics.

**Table 3:** *X*-*axis* strain as an average of gauges on P and NP sides at positions 1 and 2 after different wetting durations.

Strength	Designation	*x*-*axis* Strains (%) at Position-1 (ε_xx-1_)				*x*-*axis* Strains (%) at Position-2 (ε_xx-2_)			
		360 min	1440 min	2880 min	9600 min	360 min	1440 min	2880 min	9600 min
Low	M-0.65-1 M-0.65-2	0.0233 0.0274	0.0395 0.0445	0.0448 0.0511	0.0495 0.0593	0.0017 0.0025	0.0117 0.0153	0.0226 0.0294	0.0386 0.0448
Medium	M-0.55-1 M-0.55-2	0.0180 0.0130	0.0338 0.0261	0.0403 0.0322	0.0467 0.0378	−0.0002 0.0005	0.0040 0.0025	0.0084 0.0054	0.0171 0.0148
High	M-0.30-1 M-0.30-2	0.0095 0.0108	0.0187 0.0194	0.0217 0.0239	0.0293 0.0284	0.0003 −0.0001	0.0006 0.0003	0.0012 0.0010	0.0062 0.0040

**Table 4:** *Y*-*axis* strain as an average of gauges on P and NP sides at positions 1 and 2 after different wetting durations.

Strength	Designation	*y*-*axis* Strains (%) at Position-1 (ε_yy-1_)				*y*-*axis* Strains (%) at Position-2 (ε_yy-2_)			
		360 min	1440 min	2880 min	9600 min	360 min	1440 min	2880 min	9600 min
Low	M-0.65-1 M-0.65-2	0.0423 0.0411	0.0513 0.0488	0.0492 0.0476	0.0488 0.0491	−0.0022 −0.0027	−0.0072 −0.0071	0.0016 0.0016	0.0361 0.0522
Medium	M-0.55-1 M-0.55-2	0.0175 0.0094	0.0412 0.0393	0.0420 0.0414	0.0427 0.0422	−0.0011 −0.0007	−0.0033 −0.0037	−0.0031 −0.0045	0.0038 0.0046
High	M-0.30-1 M-0.30-2	−0.018 −0.009	0.0124 0.0293	0.030 0.0398	0.0383 0.0397	0.0006 −0.0006	−0.0016 −0.0027	−0.0032 −0.0040	0.0008 −0.0033

**Table 5:** *x*-*axis* strains (%) measured by gauges and DICM on P side at position-1 at different wetting durations.

Strength	Designation	By *x*-*axis* Gauge				By DICM Near *x*-*axis* Gauge			
		360 min	1440 min	2880 min	9600 min	360 min	1440 min	2880 min	9600 min
Low	M-0.65-1 M-0.65-2	0.0217 0.0256	0.0378 0.0432	0.0429 0.0510	0.0474 0.0619	0.0286 0.0287	0.0398 0.0437	0.0430 0.0489	0.0452 0.0596
Medium	M-0.55-1 M-0.55-2	00165 0.0122	0.0304 0.0261	0.0360 0.0319	0.0395 0.0354	0.0120 0.0203	0.0313 0.0256	0.0409 0.0362	0.0490 0.0387
High	M-0.30-1 M-0.30-2	0.008 0.0094	0.0161 0.0169	0.0185 0.0230	0.0243 0.0289	0.0093 0.0147	0.0203 0.0168	0.0221 0.0222	0.0242 0.0250

**Table 6:** Water absorption depths evaluated by DICM at different exposure times.

Strength	Specimen	Water Absorption Depth (mm)			
		t = 360 min	t = 1440 min	t = 2880 min	t = 9600 min
Low	M-0.65-1 M-0.65-2	28.33 25.91	45.38 43.20	51.00 51.68	60.09 61.97
Medium	M-0.55-1 M-0.55-2	21.11 19.63	32.24 32.05	39.29 43.01	48.68 54.21
High	M-0.30-1 M-0.30-2	14.10 14.64	21.80 21.20	24.54 24.15	25.01 25.03

**Table 7:** Water absorption rates in mortars of different strengths evaluated by DICM.

Strength	Specimen	Depth Before Deviation (mm)	Rate of Absorptions (mm/min^0.5^)			
			I_1_	R^2^	I_2_	R^2^
Low	M-0.65-1 M-0.65-2	49.09 46.50	0.988 1.063	0.9568 0.9766	0.230 0.249	0.9043 0.8447
Medium	M-0.55-1 M-0.55-2	34.02 47.22	0.699 0.733	0.9604 0.9867	0.197 0.133	0.9512 0.5776
High	M-0.30-1 M-0.30-2	23.53 23.15	0.468 0.418	0.9729 0.9560	0.021 0.030	0.7336 0.7838

**Table 8:** Mechanical properties and the water absorption characteristics determined by the DICM across different strengths.

Strength	Designation	Mechanical Properties		Water Absorption Characteristics by DICM			
		*f’_cm_* (MPa)	*E_m_* (GPa)	*I_1_* (mm/min^0.5^)	*I_2_* (mm/min^0.5^)	*Ɛ_xx_*_-*1* (2160)_ (%)	*Ɛ_xx_*_-*1* (9600)_ (%)
Low	M-0.65-1 M-0.65-2	40.52 45.24	22.02 21.72	0.988 1.063	0.230 0.249	0.0429 0.0486	0.0452 0.0596
Medium	M-0.55-1 M-0.55-2	61.33 59.21	26.08 25.04	0.699 0.733	0.197 0.133	0.0379 0.0296	0.0490 0.0387
High	M-0.30-1 M-0.30-2	83.66 87.97	34.82 36.18	0.468 0.418	0.0211 0.0292	0.0203 0.0207	0.0242 0.0250

## Data Availability

The original contributions presented in the study are included in the article; further inquiries can be directed to the corresponding author.
